# Nurses’ experiences of reporting the medical errors of their colleagues: a qualitative study

**DOI:** 10.1186/s12912-024-02092-8

**Published:** 2024-06-20

**Authors:** Farideh Namadi, Leyla Alilu, Hossein Habibzadeh

**Affiliations:** 1 Department of Medical Surgical Nursing, School of Nursing and Midwifery, Urmia University of Medical Sciences, Urmia, Iran; 2 Patient Safety Research Center, Clinical Research Institute, Department of Medical Surgical Nursing, School of Nursing and Midwifery, Urmia University of Medical Sciences, Urmia, Iran

**Keywords:** Medical errors, Truth disclosure, Nurses, Qualitative research

## Abstract

**Background:**

Medical error is a leading cause of disability and death in healthcare settings and reporting colleagues’ medical errors is one of the main strategies for medical error management and an ethical responsibility of all healthcare providers, including nurses. Most studies into reporting colleagues’ medical errors used quantitative designs while it seems that using qualitative designs can provide better insight in this area.

**Purpose:**

This study explored nurses’ experiences of reporting the medical errors of their colleagues.

**Methods:**

This qualitative study was conducted using the conventional content analysis approach. Participants were 22 hospital nurses purposively selected in 2021–2022 from different cities in Iran. Twenty-two in-depth semi-structured interviews were held for data collection. The data were analyzed via Graneheim and Lundman’s conventional content analysis and trustworthiness was maintained using the criteria proposed by Guba and Lincoln.

**Findings:**

The main categories of the study were burnout and intention to leave the profession and growth and development. The two subcategories of the first category were the experience of injury and the experience of violence and the two subcategories of the second category were sense of worthiness and sense of motivation. Moral distress was the most important experience of almost all participants.

**Conclusion:**

Nurses mostly have negative experiences in terms of reporting their colleagues’ medical errors. Negative experiences can act as the barriers to report colleagues’ errors while positive experiences can act as its facilitators. Improvement of the patient safety culture in healthcare settings and interpersonal relationships among healthcare providers can reduce the negative experiences and promote the positive experiences of reporting colleagues’ medical errors.

## Introduction

Medical error (ME) is a serious challenge and a leading cause of disability and death in healthcare settings [1]. By definition, ME is the omission of a correct action or commission of an incorrect action in planning or execution which may contribute to an adverse outcome [2]. MEs include a wide range of errors, including errors in medication prescription, surgical procedures, equipment use, and interpretation of clinical test results [2]. Despite great efforts for ME prevention, ME prevalence is still very high [1]. Estimates show that 237 MEs yearly occur in England, mostly in the area of primary care (38.4%) [3]. A study also showed that one twentieth of patients are exposed to preventable injuries [4]. Although there are no reliable data about the prevalence of ME in Iran, a study reported that ME prevalence in Iran is 50% and the most prevalent ME is medication errors [5]. Around 34% of MEs are associated with temporary disability and 6–9% of them are associated with permanent disability, while 3–20.8% of patients with ME-related injuries experience death [6, 7]. A study highlighted that almost 24,500 deaths occur per year due to MEs [8]. Another study in the United States reported that 6.3 million patients experience ME-related injuries and these injuries bear a cost of 19,571 million dollars [9].

Reporting the MEs of colleagues facilitates learning from errors [10] and reduces the prevalence of MEs. Healthcare authorities believe that all healthcare providers (HCPs) are responsible for ME reporting [11, 12]. The American Medical Association also supports the reporting of colleagues’ ME and highlights that physicians need to adhere to professional standards, have honest professional interactions, and attempt to report their colleagues’ personality problems, professional incompetence, and deception in order to facilitate their effective management [11]. Therefore, HCPs are encouraged and required to report any observed error to an authority [13]. Communicating MEs can directly and indirectly improve care quality and patient safety. The truth-telling and veracity ethical principles also require HCPs to report their errors [14, 15].

Nurses have significant role in reporting their colleagues’ MEs and preventing patient injury because they form the largest group of HCPs and have extensive relationships with different HCPs. Ethical principles also require nurses to practice based on ethical standards, namely non-maleficence, justice, accountability, and safe care provision [16, 17].

Although ME reporting is a professional norm and obligation [18], HCPs are sometimes reluctant to report the MEs of their colleagues. Reasons for such reluctance include exposure to difficult ethical conditions, exposure to challenging and unpleasant feelings, interpersonal conflicts, concern over colleagues’ involvement in legal problems, concern over damages to relationships with colleagues, probability of losing friends, fear over a sense of betrayal, and others’ negative attitudes towards those who report MEs [19–21]. Moreover, some historical norms hold that competent staff should support their colleagues and not report their colleagues’ errors [22], while professional commitment holds that this unprofessional practice may damage public trust in healthcare services. Nurses also experience moral distress and problems in their professional relationships when they report their colleagues’ errors. Personal, professional, and organizational barriers such as fear over employment loss, revenge, and colleagues’ anger [23–25], , medical paternalism, limited professional autonomy [26], unsupportive organizational culture, unfair punishments [24, 27, 28], lack of an effective reward system, and inadequate organizational support [23] also negatively affect nurses’ moral courage to report colleagues’ errors.

### Context in Iran

Despite specific guidelines for error management and great emphasis on confidential reporting of colleagues’ MEs in Iran, only a few MEs are reported mainly due to HCPs’ fear over the negative consequences of ME reporting. A study in Iran showed that only 36.8% of physicians tended to provide their colleagues with verbal warning about errors and 32.4% of nurses announced that they would report their colleagues’ MEs if they were serious errors [29].

Most studies into ME reporting in Iran were conducted using quantitative designs and were on reporting one’s own errors and there is limited information about reporting colleagues’ errors. Therefore, the present study was carried out in order to explore nurses’ experiences of reporting the MEs of their colleagues.

## Methods

### Design and paradigm

This qualitative study was carried out using the conventional content analysis approach. This approach helps obtain reliable data to create new knowledge, insight, and practical guides for action [30]. Moreover, this method uses the naturalistic paradigm to interpret meaning from textual data and helps clearly describe phenomena through concept and categories [31].

### Sampling strategy

Participants were 22 hospital nurses selected via purposive sampling. Eligibility criteria were employment as a hospital nurse, bachelor’s degree or higher in nursing, a work experience of at least two years in one ward, and agreement for participation, while voluntary withdrawal was the only exclusion criterion. The first three participants were selected through consulting the managers of the study setting. They had the experience of working in different medical-surgical care wards and patient safety committees. Other participants were selected based on the results of previous interviews to complement the developing categories and subcategories. For example, when participant 15 said that “Based on my experience of working in hospitals in small and large cities, nurses in small cities have closer relationships with hospital nursing managers and hence, ME reporting and patient safety protection in these cities are not effective”, we interviewed nurses from small cities to collect more in-depth data in this area.

### Participants

Participants were six male and sixteen female nurses (22 in total) with bachelor’s or master’s degree or PhD studentship and a work experience of 2–38 years (Table 1). They were selected from different hospital wards in East Azerbaijan, Kerman, Ilam, Kurdistan, and Sistan and Baluchistan provinces, Iran. People in these provinces have different sociocultural backgrounds. As workplace atmosphere and organization culture can influence ME reporting, we performed sampling with maximum variation to manage their influences.

**Table 1:** Participants’ demographic and occupational characteristics

Characteristics		*N*	%
Gender	Male	6	27.27
	Female	16	72.72
Age (Years)	< 35	11	50
	35–45	7	31.81
	> 45	4	18.18
Academic degree	Bachelor’s	12	54.54
	Master’s	8	36.36
	PhD student	2	9.09
Work experience (Years)	< 10	12	54.54
	10–15	7	31.81
	> 15	3	13.63
Hospital type	Private	2	9.09
	Public	20	90.90
Interview type	Face-to-face	18	81.81
	Telephone	4	18.18
Total		22	100

### Data collection methods

Semi-structured interviews were conducted to collect the data. Interviews were started with warm-up questions and continued with questions such as “What challenges do you face when MEs occur during patient care?”, “Have you ever noticed your colleagues’ MEs?”, “What do you do when you notice a colleague’s ME?”, and “What will happen if you report a colleague’s ME?” We attempted not to interfere with the process of the interview as much as possible and asked appropriate questions to avoid deviation from the aims of the study. Probing questions were also used based on participants’ responses to the main questions. These questions included “Can you explain more about it?”, “What do you mean by this?”, and “Can you provide an example to help me better understand what you mean?” Finally, we asked participants whether they wanted to mention any other point which had not been addressed during the interviews. Interview data were audio-recorded. Some participants did not consent to audio record some pieces of their interviews due to the high sensitivity of the subject of ME and its direct impact on hospital ranking, accreditation, and budget and hence, those pieces were not recorded. At the end of the interviews, we provided participants with a telephone number and asked them not to hesitate calling us for their questions. Moreover, we got their telephone numbers to make appointment with them for complementary interviews, if any, or to ask them to review the data and the findings for the purpose of ensuring the trustworthiness of the study. Participants were free to share their information through their preferred language. Twenty participants spoke Persian and two participants spoke Azerbaijani Turkish during the interviews. The coincidence of the study and the coronavirus disease 2019 also required us to conduct four interviews over telephone. The time and the location of the interviews were set based on participants’ preferences. All recorded interviews were transcribed word by word in Persian. Data collection was kept on until the data were saturated and no new data were obtained from the interviews. Saturation was achieved with eighteen interviews. Nonetheless, four interviews were conducted to ensure saturation. Data collection lasted ten months.

### Data collection instruments

The data collection instrument was an interview guide (Table 2) developed based on the authors’ experiences. Some questions were also added to the guide during the interviews. The interviews were audio-recorded using an Android smartphone.

**Table 2:** Interview guide

Questions • What do you do when you notice a colleague’s medical error? • What will happen if you report a colleague’s medical error? • What were the reactions of other nurses to your medical error reporting? • What were the reactions of the healthcare system to your medical error reporting? • What were the reactions of the colleagues who had committed the medical error to your medical error reporting? • Have you ever been silent when noticing your colleagues’ medical errors? Why? • Why do not you speak about physicians’ medical errors? • What are fears over medical error reporting?	

### Data processing

The first author listened to each interview several times and transcribed it word by word using the Microsoft Office Word. The audio and text files of the interviews were anonymized using numerical codes and the data were managed using the MAXQDA 10 software (v. 10 R 160,410; Udo Kuckartz, Berlin, Germany).

### Data analysis

The collected data were analyzed concurrently with data collection via Graneheim and Lundman’s conventional content analysis [32]. At the beginning, interview transcripts were read several times to achieve a broad understanding of the data. Each interview transcript was divided into meaning units and the units were coded. The codes were compared and were grouped in subcategories based on their similarities. Subcategories were also grouped into categories in the same way. Finally, the three authors of the study discussed and revised the subcategories and categories.

### Techniques to enhance trustworthiness

Trustworthiness was ensured via the four criteria of credibility, dependability, confirmability, and transferability [33]. Credibility was maintained using in-depth interviews, immersion in the data, prolonged engagement with the study subject matter, and member checking by four participants. Dependability was ensured through internal peer checking by the coauthors and external peer checking by two nursing faculties. During peer checking, any disagreement was resolved through discussion. Moreover, audit trailing was used to ensure confirmability, through which all steps of the study were documented. Transferability was also ensured through sampling with maximum variation concerning participants’ work experience, gender, and affiliated ward and hospital.

## Results

A total of 168 codes were developed during data analysis and were grouped into ten concepts, four subcategories, and two main categories. The main categories were burnout and intention to leave the profession and growth and development (Table 3). Most participants had negative experiences with regard to reporting colleagues’ ME and the most important concept shared by almost all participants was moral distress.

**Table 3:** The concepts, subcategories, and main categories of the study

Concepts	Sub-categories	Main categories
Deterioration of the work conditions Loss of motivation and hope Moral distress	The experience of injury	Burnout and intention to leave the profession
Verbal abuse Boycott Revenge	The experience of violence	
Effective presence Inner satisfaction	Sense of worthiness	Growth and development
Receiving reward Improvement of nurses’ professional status	Sense of motivation	

### Burnout and intention to leave the profession

Participants reported the experience of burnout and intention to leave the profession due to reporting their colleagues’ MEs. This category shows that the negative reactions of colleagues, authorities, and organization to reporting colleagues’ MEs caused participants physical and mental fatigue and reduced their ability to effectively continue their practice. The two subcategories of this category were the experience of injury and the experience of violence.

#### The experience of injury

The experience of injury referred to the negative potential and actual effects of the changes in the organization due to error reporting on nurses’ professional practice and future prospect. The three main concepts of this subcategory were deterioration of the work conditions, loss of motivation and hope, and moral distress.

##### Deterioration of the work conditions

Participants’ experiences showed that when they reported a colleague’s error, their authorities changed the workplace of the colleague from the ward to a hospital clinic or gave them less responsibilities in order to protect patient safety. This created a staff shortage in the ward which in turn increased the workload and the responsibilities of other colleagues, reduced the opportunities for leaves, deteriorated their occupational conditions, caused them burnout, and propelled them towards leaving the profession.

*We had a colleague who always mixed different antibiotics and administered the mixture to patients which caused them side effects such as vomiting and nausea. We frequently warned him not to do that; but he didn’t mind. Finally, we provided a written report to the authorities and they transferred him to the hospital clinic. Interestingly, his occupational conditions became better, while we faced staff shortage and heavier workload (P. 2).*

##### Loss of motivation and hope

Participants’ experiences showed that reporting colleagues’ MEs not only was not associated with organizational appreciation, but also reduced organizational trust in those who reported MEs. They also reported that reporting colleagues’ MEs sometimes led to the career advancement of the colleagues. These consequences caused nurses a sense of occupational decline, professional incompetence, loss of motivation, and despair.

*If you report colleagues’ errors, managers do not take appropriate actions due to the close intimacy between managers and nurses in small cities. This makes you feel that there is no place for ethical principles and professional commitment at work and hence, you gradually experience occupational decline and feel that whatever you have learned about the professional knowledge of nursing have been futile (P. 15).*

*You lose your motivation for work when you see that the occupational conditions of those colleagues who commit errors are better than yours (P. 9).*

##### Moral distress

Some participants reported indecision about reporting colleagues’ MEs due to their fear over damage to their relationships with their colleagues, particularly their close colleagues, and also due to their fear over the consequences of ME reporting. They highlighted that their final decision not to report colleagues’ MEs caused them senses of internal ambivalence, conflict, and pangs of conscience because such decision could endanger patient safety and contradicted their professional values and beliefs.

*My patient was in critical conditions with low arterial oxygen saturation and cyanosis. The monitor showed an asystole rhythm while the patient’s heart was beating. The head nurse ordered immediate epinephrine administration and resuscitation code announcement. However, I performed a rapid assessment and found that the endotracheal tube was removed. I prevented epinephrine administration and thereby, prevented an error. Basically, I had to report this event; but as the person who had committed the error was our head nurse, I couldn’t report the error and was compelled to stay silent. This silence is not pleasant because I know there was a risk of patient injury. However, reporting this error could cause me different negative consequences (P. 8).*

*I was suffering from internal conflict because my frequent verbal warnings to my colleague were futile. On the other hand, the written report of the error might cause my colleague a sense of betrayal, while I was concerned with the safety of the patients (P. 13).*

#### The experience of violence

Violence consisted of any behavior of colleagues or authorities to impose their desires on participants and thereby, cause them suppression. This subcategory had three main concepts, namely verbal abuse, boycott, and revenge.

##### Verbal abuse

Participants’ experiences showed that they faced verbal abuse, insult, or aggression when they noticed their colleagues’ MEs, told them about their MEs, or reported their MEs to the authorities.

*One of the surgeons did not use face mask during surgeries. I told him that this practice increased the risk of infection at the surgical site. But, the surgeon shouted at me by saying that I’m not at a position to question his practice and asked me not to interfere with his practice (P. 4).*

##### Boycott

Some participants reported that when they reported colleagues’ MEs, their colleagues boycotted them, did not talk to them, did not pay attention to them, took a negative attitude towards them, and asked them to change their wards.

*One day, my head nurse asked me to communicate my concerns with her. I confidentially told her about the ward conditions and the medical errors in the ward. At the end, she told my why I don’t leave her ward and highlighted that she and twenty of the colleagues were satisfied that I leave the ward because I caused them troubles (P. 21).*

##### Revenge

Participants’ experiences also revealed that in response to reporting colleagues’ MEs, colleagues attempted to take revenge on them through mistreating or labeling them. Head nurses also caused them problems by assigning heavy responsibilities to them and paying no attention to their preferred work schedule.

*One of my colleagues administered the medications of the next morning at 24:00. They labeled me ‘snitch’ due to reporting this error (P. 7).*

*When I testified in the court that my colleague had hit a patient, that colleague, our head nurse, other nurses in the ward, and even the authorities of our organization mistreated me and imposed me a very heavy work schedule. Therefore, I attempted to change my hospital; but the authorities did not accept. Finally, I decided to leave clinical settings through opting for continuing my academic education. Now, I’m a PhD student (P. 16).*

### Growth and development

This category refers to participants’ positive experiences of reporting colleagues’ MEs and indicates how nurses’ capacities and competencies improved their professional efficiency and moved them towards excellence. The two subcategories of this category were sense of worthiness and sense of motivation.

#### Sense of worthiness

This subcategory showed that nurses felt worthy and satisfied with their ability to protect patient safety. This subcategory had two main concepts, namely effective presence and inner satisfaction.

##### Effective presence

Participants reported that they felt senses of effectiveness, happiness, and power when they saw their ability to positively change the process of treatment, obtain positive treatment outcomes, positively influence their colleagues and environment, reduce the number of MEs, and improve patient safety through reporting MEs. They interpreted these positive outcomes as their effective presence at patient bedside.

*I decided to anonymously write colleagues’ errors on a piece of paper without mentioning their names and drop the paper in the error reporting box. This helped the authorities understand the existing problems and intervene to manage them. There, I understood that even a single person could make significant changes and hence I felt a sense power (P. 6).*

*I felt happy with my ability to do something for my patients (P. 3).*

*2.1.2. Inner satisfaction*: Participants’ experiences showed that their courage in protecting patient safety through reporting colleagues’ MEs despite its potential negative consequences gave them senses of inner satisfaction and pride. This reduced their fatigue and helped them feel that they were good humans.

*But, my conscience was clean. When I reported patient hitting by a colleague, I really thought that I didn’t violate the right practice and felt satisfied with myself because patients in the psychiatric ward are really innocent and defenseless (P. 16).*

#### Sense of motivation

By motivation, we mean the process in which nurses feel greater desire and interest in reporting their colleagues’ errors. The two subcategories of this category were receiving reward and improvement of nurses’ professional status.

##### Receiving reward

Participants’ experiences indicated that receiving reward, confirmation, or career advancement due to reporting colleagues’ MEs improved their motivation for ME reporting. Such motivation in turn acted as a facilitator to further ME reporting and treatment outcome improvement.

*The same colleague always says that I would have administered a wrong medication and cause tachycardia and death for the patient if Mrs. A had not been present (P. 14).*

##### Improvement of nurses’ professional status

Some participants highlighted that their ME reporting practice was associated with positive outcomes such as others’ acceptance and positive attitudes towards nurses’ knowledge, competence, and reliability and improved their trust in nurses.

*One of my colleagues wrapped a bandage for a patient in an incorrect way. When she finished her task and returned to the station, I confidentially and respectfully told her about that. Thereafter, she asked most of her questions from me (P. 22).*

*Physicians and nurses trust me. They rapidly attend to me whenever I say something [about patient care] and justify their trust and attention by referring to my preciseness at work. This gives me a great sense of acceptability (P. 5).*

## Discussion

This study was among the handful of studies into the nurses’ experiences of reporting the MEs of their colleagues. Findings indicated that burnout and intention to leave the profession were the main negative experiences while growth and development were the main positive experiences with respect to reporting colleagues’ MEs.

Deterioration of the work conditions was one of the negative experiences of participants after reporting colleagues’ MEs. This happened due to damages to participants’ relationships with their colleagues after ME reporting, transfer of the error-committing colleagues to other hospital units, aggravation of staff shortage, and increase in nurses’ workload. The American Medical Associations states that reporting colleagues’ MEs may lead to interpersonal conflicts and create an unpleasant work environment [19]. A study also indicated that the great turnover of nurses may increase the patient safety responsibilities and workload of the nurses who remain in the ward [34].

Loss of motivation and hope was another negative experience of participants in terms of reporting colleagues’ MEs. Findings showed that nurses’ inability to use their knowledge to protect patient safety, futility of ME reporting, the organizational culture of hiding errors, non-appreciation of the staff who reported MEs, and the career advancement of those who committed MEs reduced participants’ motivation and hope. In agreement with this finding, a study showed that delay in patient care due to nurses’ heavy workload or shortage of experienced nurses caused nurses senses of despair and threat to patient safety, increased their desire to leave their profession, and caused them to feel that they did not do “a good job” [34]. Another study found that the non-fulfillment of nurses’ mental needs and expectations, the non-accountability of their organizations towards nurses, and nurses’ mistrust in their authorities’ repetitive promises negatively affected their professional motivation and interest [35]. Conversely, HCPs in organizations with a great patient safety culture are more likely to like their job, do not intend to leave their profession, and consider themselves as members of a large organizational family [26].

Another negative experience of participants with respect to reporting colleagues’ MEs was moral distress because their avoidance from ME reporting was the violation of professional values while fear over the negative consequences of ME reporting reduced their ability to bravely advocate patients and protect patient rights. Physicians in another study also reported difficult ethical conditions and moral distress when they faced their colleagues’ errors [36]. Such moral distress happens because medical tradition emphasizes professional secrecy [36] and disapproves ME reporting [37], while professional commitment requires physicians to prioritize patient right and safety over personal interests. Moral distress can negatively affect nurses’ moral integrity, reduce their job satisfaction, and increase their intention to leave their organization or profession [24, 27, 38].

Our findings also indicated that participants were unable to show the necessary moral courage to report their colleagues’ MEs, particularly the errors of their senior staff or managers, due to their fear over the negative consequences of ME reporting. Previous studies also showed that fear and concern over revenge, anger, or colleagues’ negative reactions may cause indecision about ME reporting among nurses [20, 28, 38]. Another study showed that the dominant medical paternalism in healthcare settings in Iran limited the professional autonomy of nurses and reduced their opportunities to show their abilities so that none of them could report physicians’ MEs [35]. Similarly, a study reported fear over employment loss, lack of an effective reward system, limited professional power, medical paternalism, inadequate organizational support, and suppressing environment as the barriers to moral courage among nurses in Iran [23].

Verbal violence, boycott, and revenge were the other negative experiences of participants with regard to reporting colleagues’ MEs. In agreement with this finding, the American Medical Association states that ME reporting may cause different problems for ME reporters such as damages to their interpersonal relationships, loss of friends, negative emotions, negative attitudes, and rumors about them [19]. Accordingly, they may react to these violent behaviors through absence from work or intention to leave their profession [25, 39, 40].

We found that reporting colleagues’ MEs had some positive consequences such as a sense of effective presence, inner satisfaction, reward, and improvement of nurses’ professional status. Participants reported professional growth and development following adherence to professional beliefs and values, protection of patient safety, and fostering positive attitudes towards nurses among other HCPs. We could not find any study in this area for the sake of comparison. Electronic and paper-based error documentation systems in hospitals do not provide the possibility of identifying the positive emotions that HCPs experience during and after ME reporting and hence, the identification of these emotions was one of the strengths of the present study.

### Study limitations

We had to interview four participants over telephone due to the coincidence of the study with the coronavirus disease 2019. Moreover, we had to hold the interviews after participants’ work shifts because interviewing them during their shifts might cause them stress or interrupt the process of patient care.

## Conclusion

This study concludes that nurses experience different negative and positive consequences after reporting colleagues’ MEs, from burnout and intention to leave the profession to growth and development. The ineffective management of the negative consequences of reporting colleagues’ MEs may lead to negative consequences such as job dissatisfaction, job turnover, moral distress, violence, disappointment, staff shortage, unpleasant workplace environment, increased risk of MEs, more injuries to patients, and public distrust in healthcare systems. Strategies such as the promotion of a supportive ME reporting culture, appreciation of ME reporting, support for HCPs who commit errors, provision of rewards and incentives to HCPs who report MEs, positive role-modeling, employment of professional and competent managers, and improvement of interpersonal and professional relationships among HCPs are recommended to improve ME reporting in healthcare settings, reduce its negative consequences, and promote its positive consequences. These strategies can in turn reduce the prevalence of errors, facilitate learning from errors, prevent patient injury, and improve patient safety.

## Data Availability

The datasets of the study are available from the corresponding author upon reasonable request.
